# Membrane-bound estrogen receptor-α expression and epidermal growth factor receptor mutation are associated with a poor prognosis in lung adenocarcinoma patients

**DOI:** 10.1186/1477-7819-10-141

**Published:** 2012-07-11

**Authors:** Katsuhiko Shimizu, Yuji Hirami, Shinsuke Saisho, Takuro Yukawa, Ai Maeda, Koichiro Yasuda, Masao Nakata

**Affiliations:** 1 Department of General Thoracic Surgery, Kawasaki Medical School, Kurashiki, Okayama, 701-0192, Japan

**Keywords:** Membrane-bound estrogen receptor-α, Epidermal growth factor receptor mutation, Lung adenocarcinoma

## Abstract

**Background:**

The purpose of this study is to clarify the correlations between the expression of membrane-bound estrogen receptor-α (mERα) and epidermal growth factor receptor (EGFR) mutation and clinicopathological factors, especially in relation to the prognosis, in patients with lung adenocarcinoma.

**Methods:**

We conducted a retrospective review of the data of 51 lung adenocarcinoma patients with tumors measuring less than 3 cm in diameter. Immunohistochemical staining for mERα expression and detection of the *EGFR* mutation status were performed.

**Results:**

Among the 51 patients, the tumors in 15 showed both mERα expression and *EGFR* mutation. ("double positive") Significant associations between "double positive" and vascular invasion, vascular endothelial growth factor expression, and Ki-67 expression were observed. A multivariate analysis revealed that only "double positive" was an independent risk factor influencing the recurrence-free survival.

**Conclusions:**

Presence of mERα expression together with *EGFR* mutation was found to be an independent prognostic factor for survival in patients with lung adenocarcinoma, suggesting cross-talk between mERα and *EGFR* mutation.

## Background

Lung cancer is a leading cause of cancer-related death worldwide. The recent increase in interest in lung cancer appears to be attributable to the marked increase in the global prevalence of adenocarcinoma. Especially, adenocarcinoma appears to have a predilection for women, and the association of adenocarcinoma with a smoking habit may be less than that for the other histological subtypes of lung cancer [1,2]. These features of lung adenocarcinoma suggest that some factors peculiar to sex may be involved in the clinicopathology of this cancer, and some preference for female-associated pathways in the development of this form of lung cancer.

Estrogen exerts most of its effects in breast cancer via its receptors expressed in the tumor tissue; estrogen receptor (ER) α and ß. In breast cancer, the expression of ERα is a useful marker that provides information on the patient prognosis and the potential efficacy of hormone therapy [3]. Since ER α and ß are also well known to be expressed in both normal lung epithelial cells and lung cancers, a possible role of estrogen has been proposed in lung carcinogenesis [4]. Known for decades, ERα is a nuclear steroid receptor that is expressed in breast, ovarian, and endometrial tissue, but antibodies used to detect ERα in breast cancer show little or no reactivity in lung cancer tissues. On the other hand, non-nuclear (membrane-bound) ERα was described in 2002. Using this antibody that recognizes the ERα carboxy-terminus, staining was found in the cytoplasm and cell membrane [4]. This membrane-bound ERα comprises variant isoforms that lack the amino-terminus, because they cannot be detected by antibodies that recognize the ERα amino-terminus. In this study, we used this antibody for membrane-bound ERα (mERα).

The other well known female-related factor is mutation of the epidermal growth factor receptor (*EGFR*). *EGFR* tyrosine kinase inhibitors (EGFR-TKIs) produce a dramatic clinical response in a significant proportion of patients with lung cancer [5]. In 2004, response to EGFR-TKIs was ascribed to the presence of some type of gene mutations in the tyrosine kinase domain of *EGFR*[6,7]. The *EGFR* mutations in lung cancer associated with sensitivity to EGFR-TKIs occur more frequently in women, nonsmokers, Asians, and with adenocarcinomas [8,9].

Estrogen directly stimulates the transcription of estrogen-responsive genes of lung cells and transactivates the EGFR pathway. Stimulation of ER has been reported to increase the activity of the EGFR signal, and EGFR signal increases the activity of the ER [10]. Strong nuclear expression of ERß has been shown to be correlated with the presence of *EGFR* mutation, and the favorable prognostic significance of ERß expression has been shown to be influenced by the presence of *EGFR* mutation in lung adenocarcinoma [11]. However, to date, no report has described the correlation between mERα expression and *EGFR* mutation.

Based on these data from previous studies, we investigated the association between the expression of mERα and *EGFR* mutation in lung adenocarcinoma. In addition, we restricted the tumor size of the adenocarcinomas to tumors measuring less than 3 cm in diameter, because *EGFR* mutation is considered an early event in the pathogenesis of lung adenocarcinoma [12]. The purpose of this study was to clarify the correlations between the expression of mERα and *EGFR* mutation and clinicopathological factors, in relation to the prognosis of the patients. In addition, using immunohistochemistry to determine the expression of vascular endothelial growth factor (VEGF) and Ki-67, we studied the tumor proliferative activity and angiogenesis in adenocarcinomas showing mERα expression and *EGFR* mutation.

## Methods

### Study population

Fifty-one patients with lung adenocarcinoma measuring less than 3 cm in diameter, who underwent surgical resection (lobectomy or segmentectomy) with systematic lymph node dissection, at the Kawasaki Medical School Hospital between 2007 and 2009 were enrolled in this study. None of the patients had received either radiotherapy or chemotherapy prior to surgery. The histological diagnosis of the tumors was based on the criteria of the World Health Organization, and the tumor, nodule, metastasis (TNM) stage was determined according to the criteria in 2009. Written informed consent was obtained from each patient for the study of the excised tissue samples from the surgical specimens. This study was conducted with the approval of the institutional Ethics Committee of Kawasaki Medical School. Follow-up information up to recurrence, or March 2012, was obtained from medical records.

All patients underwent fluorodeoxyglucose positron emission tomography (FDG-PET) before the surgery. The PET and computer tomography (CT) examinations were performed with a dedicated PET/CT scanner (Discovery ST Elite; GE Healthcare, Japan), at 115 minutes after intravenous injection of 150 to 220 MBq of ^18^FDG (FDGscan, Universal Giken, Nihon Mediphysics, Tokyo, Japan). The regions of interest (ROI) were placed three-dimensionally over the lung cancer nodules. Semiquantitative analysis of the images was performed by measuring the maximal standardized uptake value (SUV_max_) of the lesions.

### EGFR mutation analysis

Analysis to detect *EGFR* mutations was performed in the resected, paraffin-embedded lung cancer tissues by a peptide nucleic acid-locked nucleic acid (PNA-LNA) PCR clamp method [13]. For this study, the PNA-LNA PCR clamp assay was performed at Mitsubishi Kagaku Bio-clinical Laboratories, Inc, Tokyo, Japan.

### Immunohistochemical staining

Immunohistochemical analyses were performed in the resected, paraffin-embedded lung cancer tissues. After microtome sectioning (4 μm), the slides were processed for staining using an automated immunostainer (Nexes; Ventana, Tucson, AZ, USA). The streptavidin-biotin-peroxidase detection technique using diaminobenzidine as the chromogen was applied. The primary antibodies were used according to the manufacturer’s instructions (ERα:, clone HC-20, Santa Cruz Biotechnology, Santa Cruz, CA, 1/500 dilution; VEGF:, clone A-20, Santa Cruz Biotechnology, Santa Cruz, CA, 1/300 dilution; Ki-67: clone MIB-1, Dako Cytomation, Kyoto, Japan, 1/100 dilution). The slides were examined by two investigators who had no knowledge of the corresponding clinicopathological data. The expression of each marker protein was examined and evaluated according to the original protocol reported previously.

ERα expression was categorized into eight grades according to previously described immunohistological scores [14]. Initially, six degrees of the proportional scores for positive staining were assigned according to the proportion of positive tumor cells (0, none; 1, < 1/100; 2, 1/100 to 1/10; 3, 1/10 to 1/3; 4; 1/3 to 2/3; 5, > 2/3). Next, an intensity score was assigned, which represented the average intensity in the tumor cells showing positive tumor staining (0, none; 1, weak; 2, intermediate; 3, strong). The proportional and intensity scores were then added to obtain a total score, ranging from 0 to 8. For the statistical analysis, ERα expression was judged as positive when the score was ≥ 4. VEGF expression was judged as positive when more than 20% of the cancer cell cytoplasm showed positive staining [15]. The labeling index of Ki-67 was measured by determining the percentage of cells with positively stained nuclei. Ki-67 expression was judged as positive when more than 10% of the cancer cell nuclei showed positive staining [16].

### Statistical analysis

Statistical analysis was performed for examining significant differences among the groups and possible correlations between presence/absence of mERα expression/*EGFR* mutation and the clinicopathological features using Fisher’s exact test or the chi square (*χ*^2^) test as appropriate. An unpaired *t*-test was used for comparison of the continuous data. Multivariate analyses were performed using logistic regression analysis. To explore the association between recurrence-free survival (RFS) and the presence of mERα expression/*EGFR* mutation, a Kaplan-Meier survival analysis was performed by stratifying significant predictor variables identified in the Cox proportional hazards model. All the statistical analyses were conducted using SPSS software (Version 17.0; SPSS Incorporation, Chicago, IL, USA). All statistical tests were two-sided, and probability values < 0.05 were regarded as statistically significant.

## Results

### Clinical characteristics

The characteristics of the patients are summarized in Table 1. The patients ranged in age from 46 to 83 years (mean, 66.8). There were 23 men and 28 women. The median follow-up period was 34 months (range 3 to 54 months).

**Table 1:** The patient characteristics

**Characteristics**	**Number of patients**	**%**
Age		
<70	31	60
≥70	20	40
Sex		
Male	23	48
Female	28	52
Tumor differentiation		
well	32	68
moderate	14	22
poor	5	10
Lymphnode metastasis		
negative	43	87
positive	8	13
Pathological stage		
IA	32	62
IB	11	22
II(A+B)	3	6
III(A+B)	5	10
Adjuvant chemotherapy		
Yes	13	35
No	38	65

### Relationship between mERα expression and the clinicopathological characteristics

**Figure 1:**
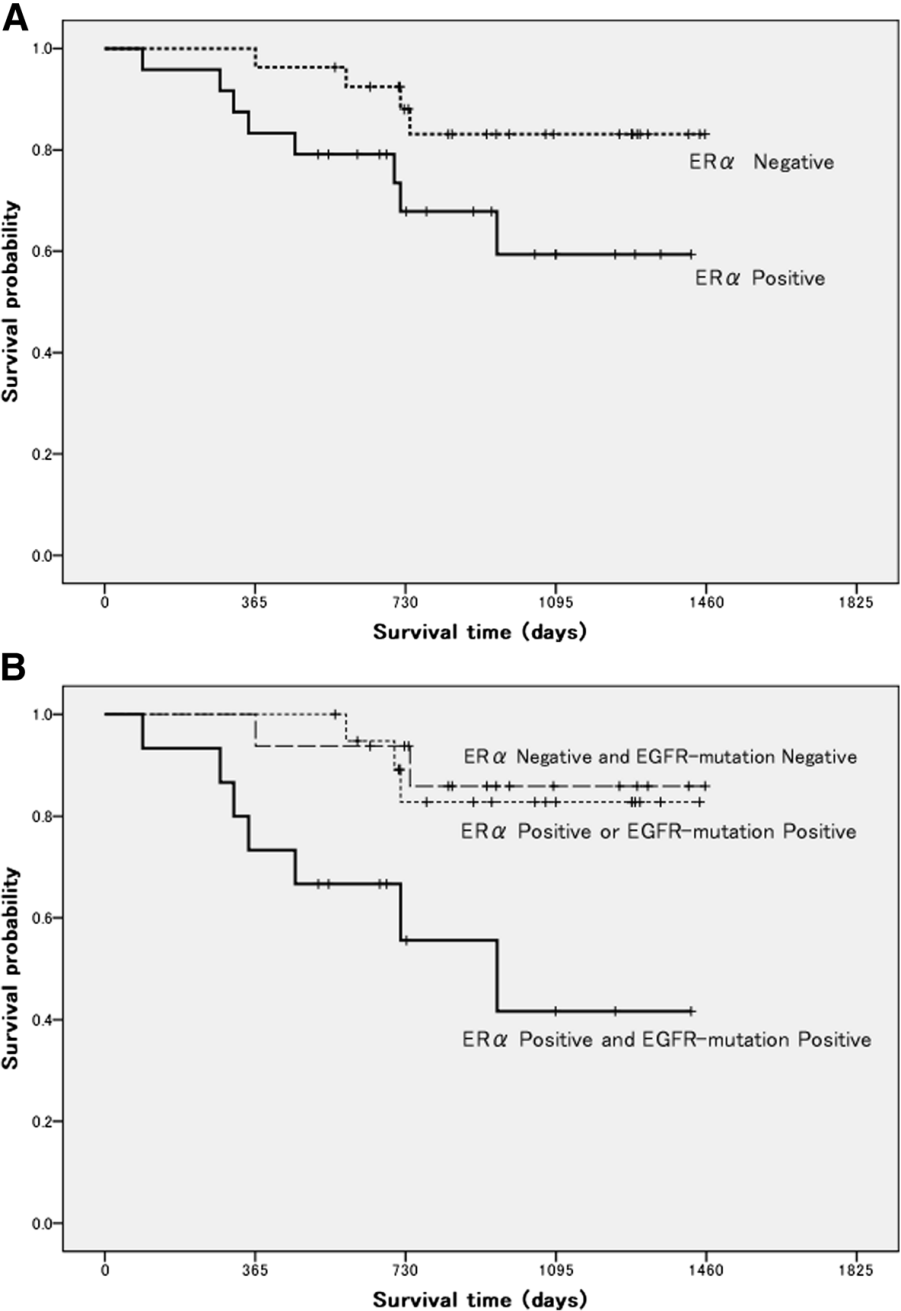
**A. Kaplan-Meier curve for recurrence-free survival according to the presence or absence of membrane-bound ERα expression.** The RFS tended to be worse in patients showing elevated mERα expression level in the tumor cells than that of the patients not showing tumor-cell mERα expression (*P* = 0.076, log-rank test). **B.** The RFS of the patients in the double-positive group was significantly worse than that of the other patients (*P* = 0.003, log-rank test).

Of the 51 patients, 24 exhibited marked increase of the immunoreactivity of the tumor cells for mERα, whereas the remaining 27 showed no increase of mERα expression. Significant associations of the mERα expression level in the tumor cells were observed with the tumor differentiation grade (*P* = 0.019), presence or absence of vascular invasion (*P* = 0.001), and the SUV_max_ on FDG-PET (*P* = 0.005), but not with age (*P* = 0.717), sex (*P* = 0.921), smoking status (*P* = 0.615) or tumor size (*P* = 0.051) (Table 2). The RFS tended to be worse in patients showing elevated mERα expression level in the tumor cells than that of the patients not showing tumor-cell mERα expression; however, the association was not statistically significant (*P* = 0.076, log-rank test; Figure 1A).

**Table 2:** **Association of membrane-bound ERα (mERα) expression / *EGFR* mutation status and clinicopathological variables**

	**mERα expression**				** *EGFR* mutation**		
**Characteristics**	**n**	**Negative**	**Positive**	**p-value**	**Mutant**	**Wild**	**p-value**
Patients, number	51	27	24		26	25	
Age(mean), year		66.6	66.4	0.717	67.5	65.4	0.391
Sex				0.921			0.036
Male	23	12	11		8	15	
Female	28	15	13		18	10	
Smoking				0.615			0.124
smoker	21	12	9		8	13	
never-smoker	30	15	15		18	12	
Tumor size(mean), mm		20.1	23.6	0.051	24.1	19.4	0.017
PET SUVmax		4.16	8.00	0.005	4.94	5.01	0.711
Tumor differentiation				0.019			0.691
well	32	21	11		17	15	
moderate/poor	19	6	13		9	10	
Vascular invasion				0.001			0.006
negative	35	21	11		13	22	
positive	16	3	13		13	3	

### Relationship between the mutation status of EGFR and the clinicopathological characteristics

Of the 51 patients, 26 had *EGFR* mutation, whereas the remaining 25 had wild-type *EGFR*. Significant associations of the *EGFR* mutation status were observed with sex (*P* = 0.036), tumor size (*P* = 0.017) and presence or absence of vascular invasion (*P* = 0.006), but not with age (*P* = 0.319), smoking status (*P* = 0.124), SUV_max_ on FDG-PET (*P* = 0.711) or tumor differentiation grade (*P* = 0.691) (Table 2).

### Associations of mERα expression and EGFR mutation with VEGF and Ki-67 expression

mERα expression was significantly correlated with VEGF expression (*P* < 0.001) and Ki-67 expression (*P* = 0.001). However, the presence of EGFR mutation was not correlated with either VEGF expression or Ki-67 expression (Table 3).

**Table 3:** **Relationship between membrane-bound ERα (mERα) expression or *EGFR* mutation and VEGF or Ki-67 expression**

	**mERα expression**			** *EGFR* mutation**		
**Factor**	**Negative**	**Positive**	**p-value**	**Mutant**	**Wild**	**p-value**
VEGF						
negative	17	2	<0.001	9	10	0.691
positive	10	22		17	15	
Ki-67						
negative	21	8	0.001	13	16	0.313
positive	6	16		13	9	

### Relationships between mERα expression, EGFR mutation and clinicopathological characteristics

We categorized the 51 patients according to the presence or absence of mERα expression and *EGFR* mutation status as follows: Group-1 (n = 15): both mERα expression and *EGFR* mutation (double-positive); Group-2 (n = 20): either mERα expression or *EGFR* mutation (single-positive); Group-3 (n = 16): neither mERα expression nor *EGFR* mutation (double-negative). Significant association of the double-positive status was observed with sex (*P* = 0.036), presence of vascular invasion (*P* < 0.001), VEGF expression (*P* = 0.018) and Ki-67 expression (*P* = 0.003), but not with age (*P* = 0.097), tumor differentiation grade (*P* = 0.150), SUV_max_ on FDG-PET (*P* = 0.168) (Table 4). The RFS of the patients in the double-positive group was significantly worse than that of the other patients (*P* = 0.003, log-rank test; Figure 1B).

**Table 4:** **Relation among membrane-bound ERα (mERα) expression, *EGFR* mutation and clinicopathological characteristics**

**Characteristics**	**mERα negative & * EGFR* wild**	**mERα positive or *EGFR* mutant**	**mERα positive & *EGFR* mutant**	**p-value**
Patients, number	16	20	15	
Age (mean), year	67.9	63.7	69.7	0.097
Sex				
Male	23	8	15	0.036
Female	28	18	10	
PET SUVmax	5.03	5.34	7.77	0.168
Tumor differentiation				0.150
well	11	17	7	
moderate+poor	5	3	8	
Vascular invasion				<0.001
negative	15	16	4	
positive	1	4	11	
VEGF expression				0.018
negative	10	7	2	
positive	6	13	13	
Ki-67 expression				0.003
negative	11	15	3	
positive	5	5	12	

A univariate analysis revealed that tumor differentiation grade (*P* = 0.006), pathological stage (*P* = 0.005) and double-positive status (*P* = 0.003) were independent risk factors influencing the RFS. However, a multivariate analysis identified only double-positive status as an independent risk factor influencing the RFS (*P* = 0.031) (Table 5).

**Table 5:** Prognostic value of recurrence-free survival

**Variable**	**Univariate analysis**		**Multivariate analysis**	
	**Unfavorable/ favorable**	**p-value**	**HR (95%CI)**	**p-value**
Sex	male / female	0.821		
Tumor differentiation	moderate+poor / well	0.006	1.96 (0.77-5.00)	0.157
Pathological stage	IB-IIIA/ IA	0.005	2.74(0.63-11.83)	0.178
double positive	Yes/ No	0.003	4.02 (1.13-14.22)	0.031

double positive: membrane-bound ERα expression positive and *EGFR* mutation positive.

*HR*: hazard ratio.

95%CI: 95% confidence interval.

## Discussion

There have been several reports of cross-talk between ER (ERα or ERß) and EGFR status (protein expression or gene mutation). This is the first report focusing on mERα and *EGFR* mutation. In the present study, we found that patients with lung adenocarcinoma who had both mERα expression and *EGFR* mutation showed significantly poorer outcomes.

One of the factors peculiar to sex reported to be involved in lung cancer development is estrogen. For example, treatment with estrogen plus progestin in postmenopausal women did not increase the incidence of lung cancer, but increased the number of deaths from lung cancer, in particular deaths from non-small-cell lung cancer (NSCLC) [17]. ER enhances transcription in response to estrogens by binding to estrogen response elements and utilizing activator protein sites [18,19]. ERα exerts an augmenting effect on cell proliferation. On the other hand, ERß exerts a suppressive effect on cell proliferation via inhibition of ERα transcriptional activity [20,21]. The differential roles of ERα and ß in lung carcinogenesis and their biological properties are still controversial. In our study, mERα expression was significantly correlated with VEGF and Ki-67 expression. Therefore, we suggest that mERα may exert an augmenting effect on angiogenesis and cell proliferation.

Some recent studies have suggested the existence of bidirectional signaling between EGFR and ER [22,23]. In addition, two clinical studies have suggested the existence of cross-talk between ER and EGFR. First, Kawai *et al*. demonstrated that the combined overexpression of mERα and EGFR protein in patients with NSCLC was predictive of poorer outcomes [24]. They showed that while overexpression of either mERα or EGFR was also predictive of poor outcomes, combined overexpression of mERα and EGFR was an independent prognostic factor, suggesting the existence of cross-talk between mERα and EGFR. Overexpression of EGFR has been observed and its prognostic significance confirmed in various cancers. In NSCLC, Salvaggi *et al*. showed that overexpression of EGFR was correlated with a poor prognosis [25]. However, the factor that is most strongly associated with from EGFR-TKI therapy has been identified as *EGFR* mutation, but not EGFR protein expression [9]. In the present study, for the treatment of patients with NSCLC, we studied *EGFR* mutation but not EGFR protein expression. Second, Nose *et al*. demonstrated that the favorable prognostic significance of overexpression of ERß was influenced by the presence of *EGFR* mutation in lung adenocarcinoma [11]. They showed that the status of *EGFR* mutation did not affect the RFS, but that ERß expression was associated with a favorable prognosis. To date, several studies have identified ER as a prognostic factor in lung cancer. In general, ERα expression seems to be associated with a poor prognosis, and ERß expression with a favorable prognosis [14,24,26-28].

An important finding of the present study was that mERα expression and the categorized status of ERα expression/*EGFR* mutation was significantly correlated with the expression of Ki-67 and VEGF. Immunostaining with the Ki-67 antibody is a widely accepted method for evaluating the proliferative activity in a variety of human tumors. Tumors showing a high expression index of Ki-67 are frequently more aggressive than tumors showing a low Ki-67 expression index [16]. On the other hand, the VEGF family of proteins modulates angiogenesis, which is essential for tumor growth and metastasis. Expression of VEGF has been shown to be associated with tumor angiogenesis, metastasis, and prognosis in several cancers, including NSCLC [15]. To the best of our knowledge, no reports to date have shown a correlation between the expression of ER and VEGF or Ki-67. Our results using tissues from patients with lung adenocarcinoma tumors measuring less than 3 cm in diameter indicate that double marker positivity was significantly correlated with the expression of Ki-67 and VEGF.

## Conclusions

This study demonstrated that the presence of mERα expression together with *EGFR* mutation is an independent prognostic factor in patients with lung adenocarcinoma, suggesting the existence of cross-talk between mERα expression and *EGFR* mutation.

## Abbreviations

CT, Computer tomography; EGFR, Epidermal growth factor receptor; EGFR-TKI, EGFR tyrosine kinase inhibitor; ER, Estrogen receptor; FDG-PET, Fluorodeoxyglucose positron emission tomography; mERα, Membrane-bound estrogen receptor; NSCLC, Non-small-cell lung cancer; PCR, Polymerase chain reaction; PNA-LNA, peptide nucleic acid-locked nucleic acid; RFS, Recurrence-free survival; ROI, Regions of interest; SUVmax, maximal standardized uptake value; TNM, Tumor, nodule, metastasis; VEGF, Vascular endothelial growth factor.

## Competing interests

The authors declare that they have no competing interests.

## Authors’ contributions

Study concept and design: KS, MN. Data acquisition: SS, AM, KY. Data analysis and interpretation: KS, TY. Statistical analysis: KS, YH. Manuscript preparation: KS Manuscript review: MN. All authors have read and approved the final manuscript.
